# Luteinizing Hormone-Releasing Hormone Distribution in the Anterior Hypothalamus of the Female Rats

**DOI:** 10.5402/2013/870721

**Published:** 2013-05-09

**Authors:** Leandro Castañeyra-Ruiz, Ibrahim González-Marrero, Agustín Castañeyra-Ruiz, Juan M. González-Toledo, María Castañeyra-Ruiz, Héctor de Paz-Carmona, Agustín Castañeyra-Perdomo, Emilia M. Carmona-Calero

**Affiliations:** ^1^Departamento de Anatomía, Facultad de Medicina, Universidad de La Laguna, Ofra s/n, 38071 La Laguna, Tenerife, Spain; ^2^Departamento de Farmacología, Facultad de Medicina, Universidad de La Laguna, Ofra s/n, 38071 La Laguna, Tenerife, Islas Canarias, Spain; ^3^Departamento de Biotecnología, Instituto de Investigación y Ciencias de Puerto del Rosario, c/Tenerife 35, 35600 Puerto del Rosario, Fuerteventura, Isla Canarias, Spain

## Abstract

Luteinizing hormone-releasing hormone (LHRH) neurons and fibers are located in the anteroventral hypothalamus, specifically in the preoptic medial area and the organum vasculosum of the lamina terminalis. Most luteinizing hormone-releasing hormone neurons project to the median eminence where they are secreted in the pituitary portal system in order to control the release of gonadotropin. The aim of this study is to provide, using immunohistochemistry and female brain rats, a new description of the luteinizing hormone-releasing hormone fibers and neuron localization in the anterior hypothalamus. The greatest amount of the LHRH immunoreactive material was found in the organum vasculosum of the lamina terminalis that is located around the anterior region of the third ventricle. The intensity of the reaction of LHRH immunoreactive material decreases from cephalic to caudal localization; therefore, the greatest immunoreaction is in the organum vasculosum of the lamina terminalis, followed by the dorsomedial preoptic area, the ventromedial preoptic area, and finally the ventrolateral medial preoptic area, and in fibers surrounding the suprachiasmatic nucleus and subependymal layer on the floor of the third ventricle where the least amount immunoreactive material is found.

## 1. Introduction

The luteinizing hormone-releasing hormone (LHRH) is a gonadotropin releasing hormone (GnRH), which acts on the pituitary hormones as a follicle stimulating hormone (FSH) and luteinizing hormone (LH), which act on the gonads, [1]. The GnRH neurons are originated in the nasal epithelium and migrate accompanying the fibers of the vomeronasal and terminal nerves [2, 3] up to the anterobasal part of the brain, where they enter the brain together with nerve terminals and then move caudally to the preoptic hypothalamus, where GnRH neurons are definitively located [2, 4]. These GnRH neurons and fibers are mainly located in the anteroventral third ventricle region, specifically in the preoptic area (PA) and the organum vasculosum of the lamina terminalis (OVLT) [5]. The anterior hypothalamus is the major region of the diencephalon implicated in the development of the olfactory system and the sexual differentiation of the brain. Most of the GnRH neurons axons project to the external zone of the median eminence where is GnRH secreted into the pituitary portal vasculature to control the release of gonadotropin [6–8]. 

The preoptic area (PA) is part of the anterior hypothalamus and is confined to the anteroventral region of the third ventricle (AV3V); the PA is divided into, the medial preoptic area (MPA) and lateral preoptic area (LPA). The MPA makes its morphological appearance at eight weeks of gestation in humans and is located in the periventricular regions of the anterior hypothalamus covering the organum vasculosum of the lamina terminalis (OVLT) [9, 10]. The MPA is generally formed by small- and medium-sized neurons whose function, among others, is related to the production of gonadotropic releasing hormone (GnRH) [9, 11]. The innervation of MPA is mainly by catecholaminergic pathways, since many noradrenergic neurons and fibers are found in the MPA, which come from different parts of the brain [9–12], but also dopamine and serotonin activity is described in the MPA [13–15]. Furthermore, structural differences between the two sexes have been described in the medial preoptic area in many species of animals and in humans, which are known as sexual dimorphism of the medial preoptic area [16–18]. 

The OVLT [1, 19] belongs to the so-called circumventricular organs (CVO) [20–22], and the CVO are characterized by the absence of blood-brain barrier [1]. The OVLT is located in the anteroventral region of the third ventricle (AV3V), contains angiotensin II (AGII), and plays a critical role in the regulation of body fluid volume and cardiovascular function. The OVLT has an important role in the plasmatic increase of AGII facilitating higher blood pressure [23]; however, the OVLT also has a lot of GnRH cells and fibers [1, 24, 25]. The high density of GnRH fibers contained in the OVLT is an atypical characteristic in the GnRH neuronal system reported many years ago [6], but it is still unexplained. Furthermore, the rostrocaudal decrease of the GnRH neurons and fibers has still not been satisfactorily clarified; this is the reason why the aim of the present work is to provide new data on the distribution of the luteinizing hormone-releasing hormone (LHRH) in the anterior hypothalamus of the female rat.

## 2. Material and Methods

Brains from five female Wistar rats from Charles River Laboratories España S.A. (Barcelona, Spain) of 6 months of age were used. Rats were kept under lighting conditions of 12:12, and food and water were provided ad libitum. Rats were sacrificed at diestrus stage [26], and before sacrifice, the body weight was taken (body weigh was 310 ± 12 grams). The rats were anesthetized with chloral hydrate, fixed by intracardiac perfusion with a solution of paraformaldehyde at 1% in phosphate buffer saline (pH 7.4), dehydrated, and embedded in paraffin under standard conditions. Brains were cut into four parallel serial coronal sections of 30 micrometer thick. The ethical committee of the University of La Laguna approved the study.

### 2.1. Immunohistochemistry

**Figure 1:**
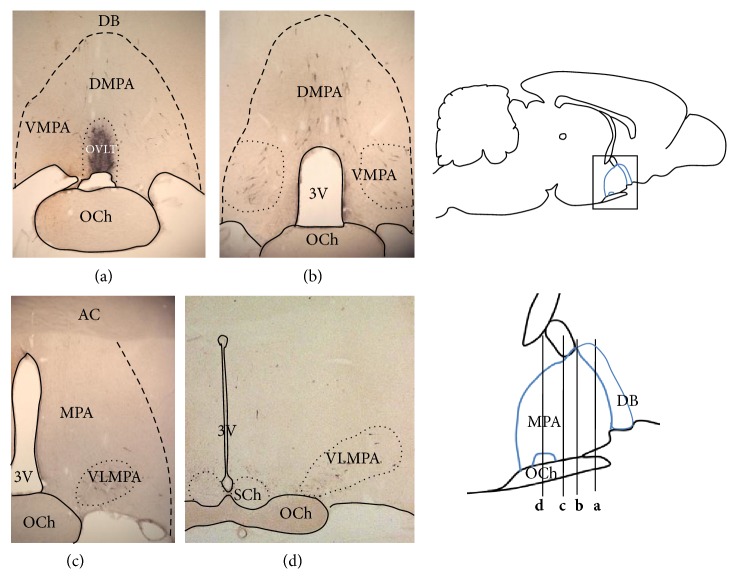
Drawing of the midsagittal rat brain and photographs showing the location of the rostrocaudal level **a**, **b**, **c**, and **d** of the coronal sections of the anterior hypothalamus.

 The sections of five coronal cephalocaudal anatomical levels of the anterior hypothalamus (Figure 1) were simultaneously incubated with a monoclonal antibody anti-LHRH (antigonadotropin-releasing Hormone antibody, Chemicon Millipore) at 1 : 500 for 24 h, followed by “DAKO” Strept ABC complex/HRP Duet, mouse/rabbit procedure. The peroxidase reaction product was visualized using diaminobenzidine intensified with nickel at 0.5% in order to get dark-blue or black immunostaining. The immunohistochemistry was also performed omitting the primary antibody in order to validate the method specificity. 

The immunohistochemistry slides were converted to digital images by using an LEICA DMRB photomicroscope with an LEICA DC 300 F camera (Germany). Image analysis was completed by ImageJ (v. 1.43 u, NIH, Bethesda, MD, USA). The “mean gray value” was measured from the selected areas for all stained tissue. This value gives the average stain intensity in grayscale units for all threshold pixels. The immunohistochemistry statistical study was conducted using the IBM SPSS statistic 19 software (one-way ANOVA).

## 3. Results

**Figure 2:**
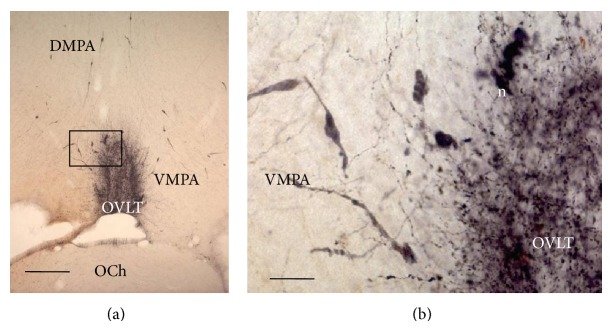
Coronal sections of the rat brain at the level **a** showing LHRH cells bodies and fibers in the OVLT, DPMA, and VPMA. A panoramic view of the PMA and OVLT; (b) magnification of (a) frame. Bar: 200 *μ*m in (a) and 20 *μ*m in (b).

**Figure 3:**
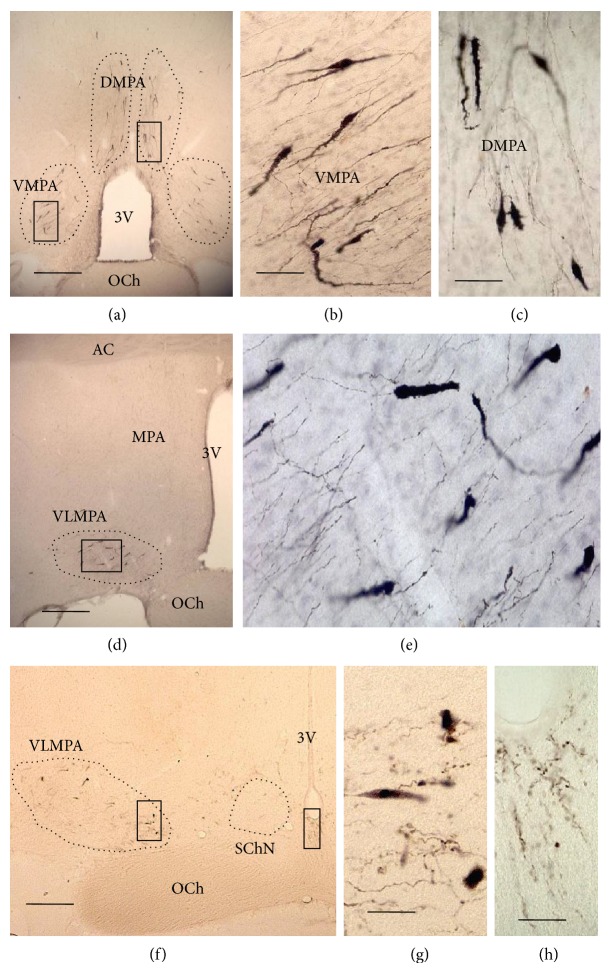
Coronal sections of the rat brain at the levels **c**, **d,** and **e** showing LHRH cells and fibers in DPMA, VPMA, and VLMPA. (a) Panoramic view of the MPA at the level **c**; (b) magnification of VMPA frame of (a); (c) magnification DMPA frame of (a); (d) panoramic view of the MPA at the level **d**; (e) magnification of the VLMPA frame of (d); (f) panoramic view of the MPA at the level **e**; (g) magnification of VLMPA frame of (f); (h) magnification of 3V floor frame of (f). Bar: 200 *μ*m in (a) and (d); 40 *μ*m in (b) and (c); 20 *μ*m in (e), (g), and (h); 150 *μ*m in (f).

Many LHRH fibers and neurons were found in different parts of the preoptic hypothalamus; the neurons presented a monopolar or bipolar morphology; the dendrites were uncomplicated, without branches and roofed with spines (Figures 2(b), 3(b), 3(c), and 3(e)). 

The distribution of LHRH cells and fibers is described with the use of the Paxinos and Watson atlas of the rat [27], the Paxinos and Franklin atlas of the mouse [28], the Allen atlas of the mouse [29], and the Hof et al. comparative cytoarchitectonic atlas of mouse brains [30], with some modifications (Figure 1). 

The antibody anti-LHRH was observed by immunohistochemistry in cell bodies and neuritis of the OVLT (Figures 1(a), 2(a), and 2(b)). Immunoreactive material was also identified in many neurons in the dorsomedial preoptic area (DMPA) and ventromedial preoptic area (VMPA), in the level **a** (Figures 1(a), 2(a), and 2(b)). Furthermore, neurons and fibers containing LHRH were located in three rostrocaudal levels **b**, **c**, and **d** (Figure 1) of the MPA located caudally to OVLT that were also subdivided into the following parts (Figures 1 and 3): dorsomedial preoptic area (DMPA) (Figures 3(a) and 3(c)), ventromedial preoptic area (VMPA) (Figures 3(a) and 3(b)), and ventrolateral medial preoptic area (VLMPA) (Figures 3(d), 3(e), 3(f), and 3(g)). LHRH fibers surrounding the suprachiasmatic nucleus (SChN) and in subependymal layer on the floor of the third ventricle were also found (Figures 3(f) and 3(h)). 

**Figure 4:**
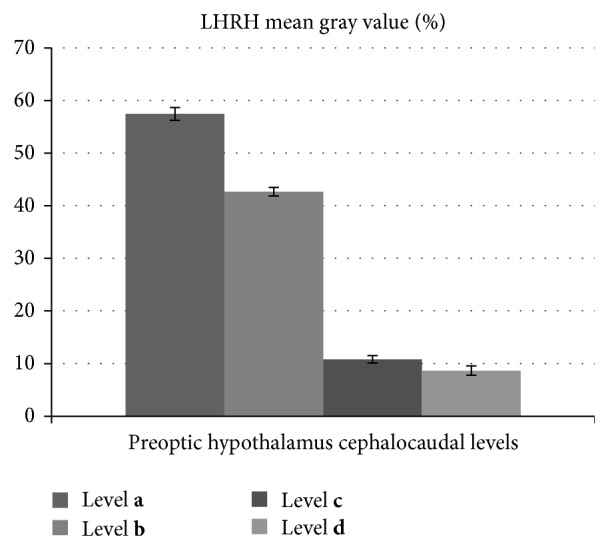
It shows densitometry of the “mean gray value” of the LHRH immunostained slides at the rostrocaudal levels **a**, **b**, **c**, and **d** of the preoptic hypothalamus. F4–14 = 1964, *P* < 0.05.

The largest amount of LHRH immunoreactive material (LHRH-ir) was found in the OVLT at the level **a** and of the MPA, after which the LHRH-ir decreased caudally (F4–14 = 1964, *P* < 0.05) till the level **d**, where the smallest amount of immunoreactive material was found (Figure 4). 

## 4. Discussion

 The findings here agree with morphological studies which report that the GnRH neurons have long and simple uni- or bipolar dendrites typically covered in spines [31, 32]. These dendrites frequently bundle with dendrites of other GnRH neurons and make close appositions that enable them to share individual synaptic input [33]. A previous study described a high density of GnRH fibers in the OVLT [6]; this was also found here where the greatest amount of the LHRH immunoreactive material was in the OVLT, but the connection and meaning of these fibers are still unexplained [8, 34].

 In view of the prevailing significance of the GnRH neurons, it is necessary to know their anatomical localization to fully understand their structure and function in general, specifically in the sexual dimorphism. At the same time, it is unclear whether the medial preoptic area is a diencephalic or a telencephalic structure because the MPA is located in the anterobasal forebrain or prosencephalon during the early stages of its development. But in the following stages, when the diencephalon and telencephalon develop and differentiate from the prosencephalon, the preoptic area is anatomically localized in the anterior hypothalamus [9, 11], although this area may be called a “residual forebrain or residual prosencephalon (RP)”, since the RP, which is rostrally limited by lamina terminalis, would be the part of the forebrain that was never differentiated as a diencephalic or telencephalic part, and when the RP develops and differentiates, the RP embraces the MPA and the OVLT. Therefore, this part of the brain has various names such as anteroventral region of the third ventricle (AV3V) [1] and septopreoptic area (POA) [35], but, in different mouse and rat atlases, this part of the anterior hypothalamus is also called and subdivided into medial preoptic area, medial preoptic nucleus, median preoptic nucleus, anteroventral periventricular nucleus, and organum vasculosum of the lamina terminalis [27–30].

 In general, this zone is referred to as the medial preoptic area (MPA), and therefore, this is the terminology used in this study. Positive LHRH cells and fibers located in different parts of MPA and in OVLT such as dorsomedial preoptic area (DMPA), Figures 1(a), 2(a), and 3(a), that would correspond to the median preoptic nucleus (MnPO) described in the mouse and atlas of Paxinos and Franklin [28]; ventromedial preoptic area (VMPA), Figures 1(a), 2(a), and 3(a), that would correspond to the preoptic medial area (MPA) described in the mouse atlas by Paxinos and Franklin [28], but to the medial preoptic nucleus of the mouse atlas by Hof et al. [30] and the median preoptic nucleus of the Allen atlas of the mouse brain [29]; OVLT, Figures 1(a) and 2, is denominated anteroventral periventricular nucleus and OVLT in the Paxinos and Franklin mouse atlas [28]; ventrolateral medial preoptic area (VLMPA) in the levels **c** and **d** (Figures 1, 3(c), and 3(d)), would correspond to the ventrolateral preoptic nucleus in the Paxinos and Franklin mouse atlas [28] and the Paxinos and Watson rat atlas [27] and the anteroventral preoptic nucleus in the mouse atlas by Hof et al. [30].

In view of that described above, one could conclude that the exact denomination of the LHRH localization is peculiar; however, one could say that the greatest amount of LHRH immunoreactive material (LHRH-ir) was found in the OVLT and decreased cephalocaudally in the preoptic hypothalamus. LHRH immunoreactive material was found in the following parts: dorsomedial preoptic area, ventromedial preoptic area, and ventrolateral medial preoptic area, and in fibers surrounding the suprachiasmatic nucleus and subependymal layer on the floor of the third ventricle where the least amount of LHRH fibers and neurons was found.

## Abbreviations

AV3V: Anteroventral region of the third ventricle
ANOVA: Analysis of variance
CVO: Circumventricular organs
DMPA: Dorsomedial preoptic area
FSH: Follicle stimulating hormone
GnRH: Gonadotropic releasing hormone
LH: Luteinizing hormone
LHRH: Luteinizing hormone-releasing hormone
LPA: Lateral preoptic area
MnPO: Median preoptic nucleus
MPA: Medial preoptic area
OCh: Optic chiasm
OVLT: Organum vasculosum of the lamina terminalis
PA: Preoptic area
POA: Septopreoptic area
RP: Residual forebrain or residual prosencephalon
SCh: Suprachiasmatic nucleus
VMPA: Ventromedial preoptic area
VLMPA: Ventrolateral medial preoptic area
3V: Third ventricle
AC: Anterior commissure
DB: Diagonal band
DMPA: Dorsomedial preoptic area
n: Neurons.
